# Unexpected Toxicity of Green Tea Polyphenols in Combination with the *Sambucus* RIL Ebulin

**DOI:** 10.3390/toxins12090542

**Published:** 2020-08-22

**Authors:** M. Ángeles Rojo, Manuel Garrosa, Pilar Jiménez, Tomás Girbés, Verónica Garcia-Recio, Manuel Cordoba-Diaz, Damián Cordoba-Diaz

**Affiliations:** 1 Area of Experimental Sciences, Miguel de Cervantes European University, 47012 Valladolid, Spain; marojo@uemc.es; 2 Area of Histology, Faculty of Medicine and INCYL, University of Valladolid, 47005 Valladolid, Spain; garrosa@med.uva.es; 3 Area of Nutrition and Food Sciences, Faculty of Medicine, University of Valladolid, 47005 Valladolid, Spain; pilarj@bio.uva.es (P.J.); girbes@bio.uva.es (T.G.); 4 Area of Pharmaceutics and Food Technology, Faculty of Pharmacy, Complutense University of Madrid, 28040 Madrid, Spain; vgrecio@ucm.es (V.G.-R.); mcordoba@farm.ucm.es (M.C.-D.); 5 University Institute of Industrial Pharmacy (IUFI), Complutense University of Madrid, 28040 Madrid, Spain

**Keywords:** green tea polyphenols, *Sambucus ebulus*, ebulin f, RIP, lectin, ribosome-inactivating protein, ricin

## Abstract

The safety of concentrated food complements intake is a major health concern. It has been well established that green tea polyphenols (GTPs) consumption promotes healthy effects. However, the ingestion of large amounts of GTPs is a matter of controversy due to reported adverse effects. We underwent a preliminary exploration of the effects of the oral administration of a standardized concentrated GTPs preparation on mice which suffered from reversible intestinal derangement promoted by sublethal amounts of the antiribosomal lectin ebulin f from dwarf elder (*Sambucus ebulus* L.). Neither independent oral administration of 30 mg/kg body weight Polyphenon 60 nor intraperitoneal administration of 2.5 mg/kg body weight ebulin f triggered lethal toxicity. In contrast, the simultaneous administration of these same doses of both Polyphenon 60 and ebulin f triggered an important and unexpected synergistic toxic action featured by the biphasic reduction of weight, which continued after eight days, reaching a reduction of 40%. Lethality appeared 2 days after the onset of the combined treatment and reached more than 50% after 10 days.

## 1. Introduction

The benefits of green tea polyphenols consumption have been extensively studied and scientifically established [1,2,3,4]. Green tea polyphenols (GTPs) display specific beneficial effects on health and pathologies, especially in cardiovascular disorders and cancer [5,6], as well as in inflammatory bowel diseases (IBD), especially at low doses [7]. GTPs are highly active molecules which trigger antagonistic effects depending on the concentration used, acting as prooxidant [8] or antioxidant [6] agents. Among the most important effects described in the literature are the anti-inflammatory [9], antioxidant [10], anticancer [6,11,12,13], and antibacterial biofilm process actions [14]. The protective effect of green tea extracts against oxidative damage is related to their catechin composition [15]. Among the green tea catechins, epigallocatechin gallate (EGCG) has been by far the most studied. EGCG acts on targets, for instance, on very complex mechanisms like cancer through apoptosis of cancer cells [16,17]. The proapoptotic effect of EGCG can potentiate the action of drugs like sulindac, which promotes apoptosis in rat colon cells, thus leading to an improvement of the therapeutic efficiency with the reduction of aberrant crypt foci [12]. These beneficial actions of green tea polyphenols on health led to the preparation of concentrated extracts enriched in catechins, to their use as dietary supplements to increase the antioxidant status of the cells and tissues, and to their use as chemo-preventive agents [18]. However, in contrast with the absence of adverse effects at moderate and regular consumption, the uncontrolled ingestion of such concentrated extracts of polyphenols could trigger adverse effects, such as those recently reviewed [19,20]. In fact, controversial results have been obtained which seem to be dependent on the animal species, gender, age, and green tea preparation [21,22,23]. The bioavailability of GTPs could be substantially modified depending on the route of administration, or dosage form among other factors that should explain these differences [6].

High doses of (-) epigallocathechin-3-gallate trigger hepatotoxicity in mice [19]. In contrast, no subacute toxicity of green tea extract was seen in mice [18]. These discrepancies could also be due to the health condition of the animals.

Ribosome-inactivating proteins (RIPs) are enzymes widespread throughout the plant kingdom and have been suggested to form part of the plant defense system against predators like insects [24], pathogen-like viruses [25], and fungi [26,27,28,29,30]. They display N-glycosidase activity on nucleic acids [31]. Interest in RIPs derives from their toxicity and from their possible use in anticancer immunotoxins and conjugates [30,32,33,34]. Some of these RIPs show lectin properties, being known as ribosome-inactivating lectins (RILs), such as nigrin b from elderberry bark [35] and ebulin f from dwarf elder fruits [36,37]. Both nigrin b and ebulin f display dose-dependent toxicities depending on the different administration way [35,37,38,39]. Both nigrin b and ebulin f administration promoted apoptosis of intestinal crypt cells in mice, as revealed by histological analysis [38,39,40]. This effect influences the uptake of nutrients like vitamin B6 [41]. There are several animal IBD models, and as well as cultured cell IBD assays, to perform preliminary biopharmaceutic experiments with new drugs or nutrients. The *Sambucus* RILs IBD model has been proposed as an interesting alternative to classical DNBS or DSS models, since *Sambucus* RILs derangement of the bowel mucosa is reversible [41,42].

Jimenez and coauthors demonstrated that the independent administration of high oral doses of Polyphenon 60 or high intraperitoneal doses of nigrin b to mice did not affect survival. Surprisingly, the coadministration of both substances resulted in the death of some animals with serious injury on small intestine and liver [43]. Whereas nigrin triggers specific intestinal derangement, ebulin affects not only the intestinal system, but also the lungs, kidneys, heart, and spleen [40]. The aim of the present research was to characterize the GTPs composition of several aqueous green tea extracts and to evaluate their potential healthy effect in the *Sambucus ebulin* IBD model.

## 2. Results

**Figure 1:**
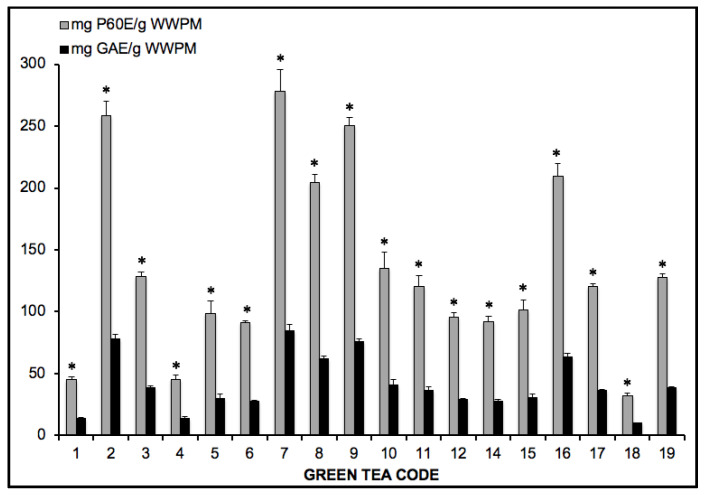
Comparative analysis of the green tea extracts solutions using gallic acid and Polyphenon 60^®^. Results expressed as mg equivalent of standard per gram (wet weight) of plant material (WWPM). * One-way ANOVA significant differences (*p* < 0.001) F value ≥ 7.661.

Analysis of the green tea extracts solutions proved that gallic acid reactivity corresponds to 3.1-times that of Polyphenon 60^®^ (Figure 1).

**Figure 2:**
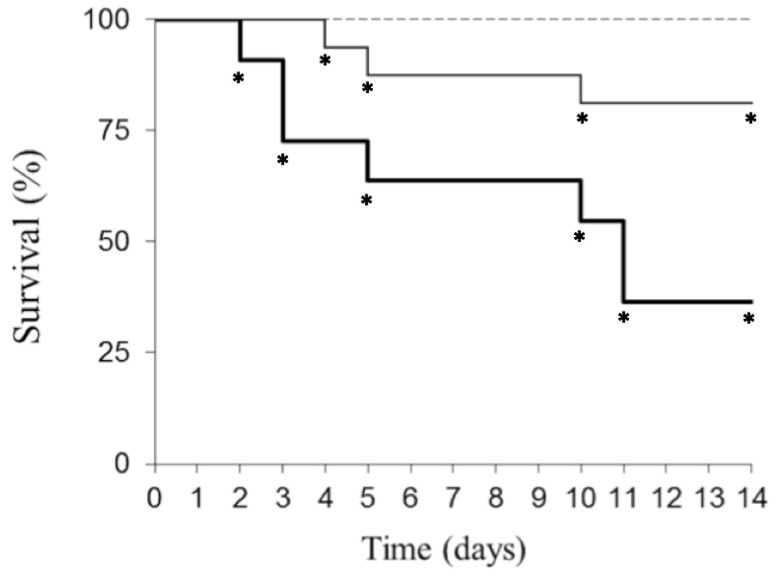
Survival evolution followed up to 14 days of mice treated with Pol60 and ebulin f. Female mice treated p.o. with 30 mg/kg body weight of Pol60 (dashed line; *n* = 7), 2.5 mg/kg body weight of ebulin f (continuous line; *n* = 16) or both together (bold line; *n* = 11). * Tukey post-hoc significant difference from Pol60 group (*p* < 0.05).

Since Polyphenon 60^®^ has a defined composition and as a previous step to the oral administration of the GTPs characterized green tea extracts, a standard solution of 30 mg/kg body weight of Pol60 were orally administered to Swiss mice with neither visible damage nor reduction of the body weight (Figure 2).

That concentration falls into the range of those used in human beings with alleged healthy results [44]. Intraperitoneal administration of 2.5 mg/kg ebulin f reduced by 20% the survival of mice 14 days after the experiment (Figure 2). The first death was registered at the fourth day and the last one at the tenth day. Unexpectedly, the combined administration to mice of both oral 30 mg/kg Pol60 and i.p. 2.5 mg/kg ebulin f notably increased toxicity of the ebulin f, resulting in a reduction of 70% of the mice survival, with the first death observed at the second day and the last one at the eleventh day (Figure 2).

**Figure 3:**
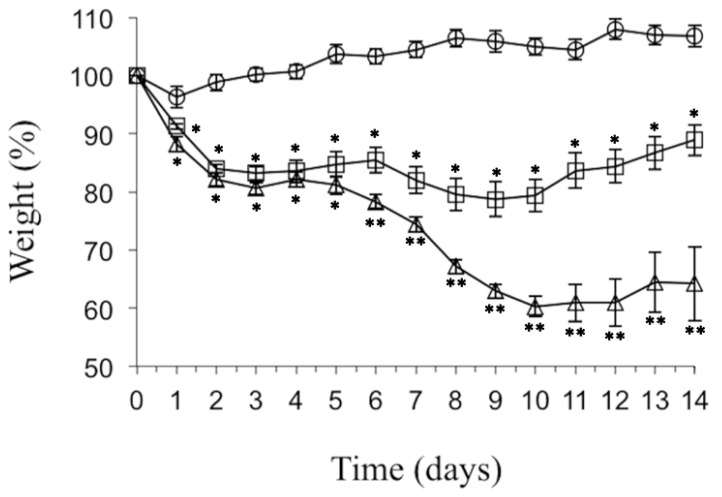
Body weight evolution followed up to 14 days of mice treated with Pol60 and ebulin f. Female mice were treated i.p. with 30 mg/kg body weight of Pol60 (circles; *n* = 7), 2.5 mg/kg body weight of ebulin f (squares; *n* = 16) or both together (triangles; *n* = 11). * significant difference from Pol60 group. ** significant difference from ebulin f group (*p* < 0.05, 1-way ANOVA with post-hoc comparison by Tukey’s test).

Concerning to the body weight changes, ebulin f alone treatment promoted a 20% reduction in the body weight three days after its administration, then showed a further small reduction and later started to recover (Figure 3). One-way ANOVA showed significant differences from the Pol60 group at all sampling times. The animals treated with both ebulin f (i.p.) and Pol60 (p.o.) suffered the reduction of body weight, and also with a biphasic pattern. The first phase of the weight loss was not significantly different, with ebulin f reaching 20% (days 1–5). The second part was larger than the observed with ebulin f alone (days 6–14), reaching 40% (Figure 3).

**Figure 4:**
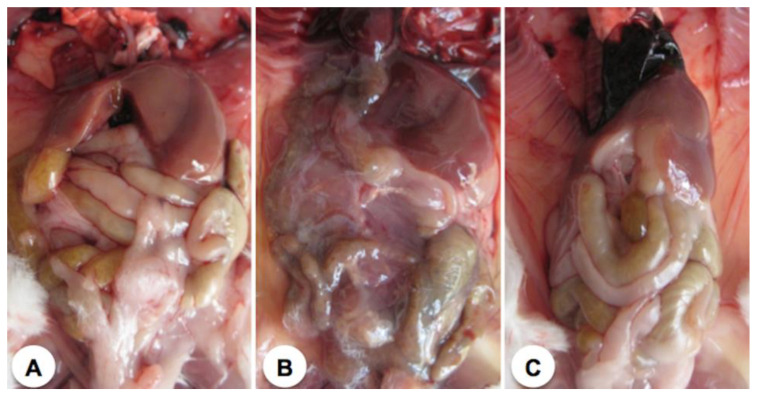
Open abdominal cavities of representative mice 11 days after treatment. (**A**) Control animal; (**B**) Animal treated i.p. with 2.5 mg/kg body weight of ebulin f; (**C**) Animal treated orally with 30 mg/kg body weight of Pol60.

Animals affected by the combined action of ebulin f and Pol60 were opened and examined. The abdominal cavities of control mice, ebulin f-treated mice, and Pol 60-treated mice euthanized at the 11th day after treatment are shown in Figure 4.

**Figure 5:**
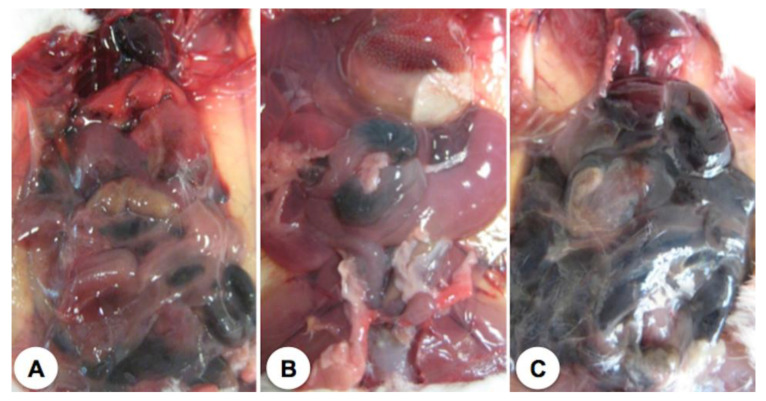
Open abdominal cavities of representative dead mice. (**A**) Animal treated i.p. with 2.5 mg/kg body weight of ebulin f, which died at the fifth day of the experiment; (**B**) Animal treated i.p. with 2.5 mg/kg body weight of ebulin f and 30 mg/kg body weight of Pol60 orally, which died at the third day of the experiment; (**C**) Animal treated i.p. with 2.5 mg/kg body weight of ebulin f and 30 mg/kg body weight of oral Pol60, which died at the eighth day of the experiment. All animal cavities were opened and photographed just after dead.

Only ebulin f-treated mice showed some alteration in the small intestine but showed a rather unaffected liver. Figure 5 shows the abdominal cavities of animals which died as a consequence of the different treatments. Ebulin-f-treated animals, which died at day five, showed an aspect very similar to those treated with ebulin f and Pol60, which died at day three (Figure 5). In this last case, the liver seems to be largely affected. The cavity of animals treated with ebulin f plus Pol60, which died at day eight, featured very dark areas in the internal organs, particularly the liver, intestines, and kidneys, presumably due to bleeding, and a weak consistency of tissues, which were difficult to remove or merely to displace in intact form. It was also characteristic of the effect the mucilage consistency and the general appearance of the organs.

## 3. Discussion

Numerous reports highlight the scientifically established benefits of green tea consumption [10,13,45]. However, several detrimental effects associated to the ingestion of large amounts of green tea or preparations of green tea highly enriched in polyphenols have been reported [19,20,46,47]. Of particular concern is the hepatotoxicity, presumably resulting from the ingestion of concentrated green tea extracts [19,48,49,50]. In experiments carried out with isolated mouse hepatocytes, it was found that high concentrations of EGCG promoted the production of reactive oxygen species and cytotoxicity [21]. Recent data indicate that intake of 1% GTPs diet aggravated colitis promoting colon carcinogenesis in mice treated with sodium dextran sulfate to induce colitis, while it decreased the activities of superoxide dismutase and catalase in nontreated mice [51]. Furthermore, intake of high-dose GTPs induces nephrotoxicity in mice suffering sodium dextran sulfate-colitis by a mechanism which involves the downregulation of antioxidant enzymes and the expression of HSP70, HSP27, and HSP90 heat-shock proteins [22]. In this line of disruption of cellular mechanisms promoted by high-dose GTPs intake, it has been argued that the prooxidant activity of polyphenols could play a significant role in the destruction of transformed cells by the activation of apoptotic mechanisms leading to tumor reduction [11].

The i.p. administration of 5 mg/kg body weight of ebulin f to mice leads to significant structural alterations consisting in atrophy of intestinal crypts, which is presented an apoptotic-like morphology and consequently leads to death of mice in 3-4 days [37,39]. In contrast, the intraperitoneal administration of 2.5 mg/kg doses to mice provoked lower mortality ([39], present results). However, as seen in Figure 2, with 2.5 mg/kg body weight dose, some lesions were produced, which had an important impact on the health of animals given orally 30 mg/kg GTPs. In fact, the derangement promoted by the combined treatment of ebulin f and Pol60 suggests a synergistic action that is more evident one week after the onset of treatment. That delayed toxicity would be the result of the combination of apoptotic actions of ebulin f preparing the target cells and GTP enhancing the apoptotic effects.

GTPs have been described also as potent prooxidant species both in vitro and in vivo [4]. They promote the formation of free radicals, which could trigger some of the apoptotic effects seen in the cancer chemoprevention [8,16,21,52,53]. The present findings support the emerging belief that consumption of large amounts of green tea polyphenols could promote some adverse effects [20]. On these grounds, we hypothesized that the proapoptotic actions of GTPs and ebulin f are synergistic, and this phenomenon is especially evident in mice bearing intestinal ebulin f-dependent injuries. Wang and coauthors recently demonstrated that high doses of EGCG suppressed antioxidant enzymes but activated the nuclear factor erythroid 2-related factor 2 (Nrf2) system [54]. A plausible explanation of this synergic effect may be that ebulin f could inhibit the synthesis of Nrf2-dependent cytoprotective enzymes, which could enhance the toxicity. Further research in the frame of quantification of NAD(P)H:quinone oxidoreductase 1, heme oxygenase 1, or glutathione S-transferase is required to demonstrate this hypothesis.

From a healthcare point of view, that synergy could be very important, for instance, in patients suffering from intestinal derangement promoted by drug treatments. In that line, our results are in agreement with the results obtained with the combined treatment of green tea polyphenols and sulindac in azoxymethane-treated rats [12]. Research is being conducted to extend our results and to gain details on the molecular basis and its histological projection of the effects reported here.

## 4. Conclusions

The oral intake of a concentrated mixture of green tea polyphenols (Pol60) by mice potentiated the toxic effects promoted by the intraperitoneal administration of ebulin f. The delayed lethality promoted by the combined treatment raised the concern of the uncontrolled ingestion and abuse of these dietary supplement preparations. Our findings pose the question of the abuse of concentrated green tea extracts as a food antioxidant supplement, in particular, when therapeutic drugs that affect the gastrointestinal tract are used or toxic compounds are ingested.

## 5. Materials and Methods

### 5.1. Materials

Polyphenon 60 (Pol60) was purchased from Sigma-Aldrich Química SA (Tres Cantos, Spain). According to its technical product data sheet, Pol60 contains (-) epicatechin-3-gallate (21.0%), (-) epicatechin (7.3%), (-) epigallocatechin (7.9%), and (-) epigallo-catechin-3-gallate (29.2%), and was dissolved freshly in type I water obtained in a Milli-Q filtration system (Millipore, Bedford, MA, USA) and used immediately to avoid oxidation. Except water, all the other reagents and solvents used were of analytical grade and purchased from Sigma-Aldrich Química S.A. (Tres Cantos, Spain).

### 5.2. Isolation of Ebulin F

The toxin ebulin f present in fruits of dwarf elder (*Sambucus ebulus* L) was prepared as described elsewhere [36,39] with some modifications. Briefly, 200 g of green dwarf elder collected in the late spring were ground to obtain a finely cut material. That material was extracted overnight with 800 mL of 280-mM NaCl containing 5-mM sodium phosphate (pH 7.5) solution, strained through cheesecloth. The fluid was then centrifuged twice at 7500× *g* for 30 min at 4 °C. The clear supernatant was filtered through a two-layer of filter paper and chromatographed through acid-treated Sepharose 6B (AT-Sepharose 6B) to obtain D-galactose-binding proteins, which included SELfd and ebulin f. Both proteins were separated by Superdex 75 chromatography with 400-mM NaCl and 5-mM sodium phosphate (pH 7.5) buffer. Fractions containing SELfd (moving faster) and ebulin f were collected avoiding cross-contamination and concentrated with Amicon (Y10 membrane) to 2–2.5 mg/mL and stored as 0.1 aliquots at −20 °C. The purity of both proteins was checked by SDS-PAGE (Figure S1).

### 5.3. Green Tea Aqueous Extract Preparation

Tea extracts were prepared according to the manufacturer’s instructions of time and temperature (Table 1) by mixing 1 g of the corresponding green tea samples with 50 mL of water. Tea extracts were used immediately for the assays.

### 5.4. Subjects

A total number of 37 Swiss female mice were housed individually in plastic cages and fed *ad libitum* with the rat/mouse maintenance, 15-mm diet (Ssniff spezialialdiäten GmbH, Soest, Germany), and with free access to water under a 12-h light-dark cycle. They were weighted daily and observed for any behavioral alteration and external signs of derangement. Handling of the animals followed the European Communities Council guidelines (2010/63/EU) and the Ethical Committee of the Animal Research and Welfare Service of the University of Valladolid (Approval Code: 607193, Date of approval: 13 November 2013).

### 5.5. Treatment

Four groups of animals were established. Group 1 (*n* = 16) was treated intraperitoneally with 2.5 mg/kg body weight of ebulin f administered as solution in 0.1-M phosphate-buffered saline, pH 7.4, in a volume of 100 µL. Group 2 (*n* = 7) received one oral (p.o.) dose of Pol60 slowly as a water solution with a blunted end needle attached to a 1-mL syringe directly to the deepest part of the stomach, avoiding any resistance of the esophagus and the pass of the liquid to the lungs. Group 3 (*n* = 11) was administered with both treatments at the same day. Finally, Group 4 (*n* = 3) consisted of littermates which received no treatment to serve as controls. Both the food pellets and water were placed close to mice on the floor of the cage.

### 5.6. Phenolic Analysis

The Folin–Ciocalteu method is a simple, fast, and robust assay of performing total phenolics content and it is routinely practiced in laboratories testing food and plant extracts. However, the main drawback of that method is that reducing agents, such as ascorbic acid or certain amino acids, can interfere with the chromophore and thus overestimate the content of phenolic compounds [55]. It has been previously reported that galloyl groups from phenolic compounds can react with each Fe(III) ion to form a stable octahedral complex, allowing each phenolic compounds molecule to react with several Fe(III) centers to form a cross-linked film [56]. The coordination between Fe(III) and galloyl groups from phenolic compounds is pH-dependent, which can be attributed to transitions between mono-, bis-, and tris-complex states [57,58]. For that reason, an alternative analytical method for the rapid and inexpensive quantification of total phenolic substances with iron (III) chloride has been developed and validated according to the ICH recommendations, based on the following criteria: Linearity, precision, accuracy, selectivity, sensitivity, and robustness. Polyphenon 60^®^ or gallic acid were used as standards (Figure S2 and Tables S1–S3).

### 5.7. Statistics

The analysis of data was conducted with SPSS software, version 25.0 (SPSS Science, Chicago, IL, USA). All data were expressed as mean ± standard deviation of three replicates unless otherwise stated. A *p* value < 0.05 was considered to be statistically significant using the Student’s *t*-test between the two means for the unpaired data. Goodness of fitting was determined by the analysis of residuals and Durbin-Watson test, as well as Shapiro–Wilks and Akaike AIC stats. One-way ANOVA and post-hoc Tukey pairwise comparisons were used to test statistical differences in the comparative analysis of the green tea extracts solutions using gallic acid or Polyphenon 60^®^.

## Figures and Tables

**Table 1:** Extraction conditions proposed by the manufacturers for the assayed green teas.

Time	Tea Name	T (°C)	t (min)
1	Kukicha 3 years	90	5
2	Sencha	90	2
3	Kukicha	90	5
4	Hojicha 3 years	100	2
5	Bancha	80	3
6	Bancha leaf	80	3
7	Matcha Second	100	0.5
8	Gyokuro	100	2
9	Matcha First	100	0.5
10	Green Salvage	90	3
11	Special Gunpowder	90	3
12	Bi Luo Chung	90	3
13	Long Jing	90	2
14	Tai Ping Hou Kui	88	1
15	Chun Mee	100	2
16	Jade Rings	90	3
17	Mo Li Feng Yan	85	3
18	Long Jing Dragon Well	90	3
19	Kukicha 3 years	90	5

## Supplementary Materials

The following are available online at https://www.mdpi.com/2072-6651/12/9/542/s1, Figure S1: SDS-PAGE of SELfd 6 µg (line 2) and ebulin f 6 µg (line 3) purified from S. ebulus fruits, Figure S2: Correlation plot between gallic acid and Polyphenon 60^®^. Table S1: Linearity. Multiple regression results, Table S2: Main precision (repeatability and intermediate precision) and robustness results obtained at three different concentration levels: low, medium and high (L/M/H), Table S3: Statistical analysis and accuracy results

## Abbreviations

DNBS	dinitrobenzene sulfonic acid
DSS	dextran sulfate sodium
RIP	ribosome-inactivating protein
IBD	inflammatory bowel diseases
EGCG	epigallocatechin gallate
GTPs	green tea polyphenols
p.o.	per os
i.p.	intraperitoneal
Pol60	Polyphenon 60
RILs	ribosome-inactivating lectins
