# Supplementary material for: Unexpected Toxicity of Green Tea Polyphenols in Combination with the Sambucus RIL Ebulin
Source: Toxins (Basel). 2020 Aug 22;12(9):542. doi: 10.3390/toxins12090542 (PMC7551510; doi:10.3390/toxins12090542)
Supplement: Supplementary file 1 [file toxins-12-00542-s001.pdf]

Supplementary Materials: Unexpected Toxicity of Green Tea Polyphenols in Combination with the *Sambucus* Ril Ebulin

María Ángeles Rojo, Manuel Garrosa, Pilar Jiménez, Tomás Girbés, Verónica Garcia-Recio, Manuel Cordoba-Diaz and Damián Cordoba-Diaz

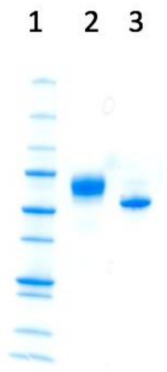

Figure S1. SDS-PAGE of SELfd 6 µg (line 2) and ebulin f 6 µg (line 3) purified from S. ebulus fruits. Markers from top to bottom (line 1): SNAI (Mr 136 kDa), BSA (Mr 68 kDa), Nigrin b (Mr 58 kDa), ovalbumin (Mr 45 kDa), SNAIV (Mr 30 kDa) and trypsin inhibitor (Mr 20 kDa).

VALIDATION OF THE SPECTROPHOTOMETRIC METHODOLOGY

Linearity. Standard solutions of 33, 67, 100, 133, 167, 200, 233, 267 µg mL^{-1} of Polyphenon 60® were prepared by diluting the stock solution with type I water. Three calibration curves were prepared from each stock solution. Curves were generated by plotting absorbance versus concentration. Linearity was evaluated by linear regression using ANOVA.

Before performing regression, the homoscedasticity of the calibration standards was verified using a Cochran´s test. The test statistic values obtained were smaller than the critical value; therefore, the variances of the calibration standards can be considered to be homoscedastic and ordinary least squares can be used to estimate the regression lines.

The plotted calibration curve, correlation coefficient above 0.99 and good relative standard deviations of response factors (coefficient of variation 1.17%) confirmed that they were linear over the concentration range assayed (Table S1).

Table 1. Linearity. Multiple regression results.

**Correlation coef. [R]**	0.99888					
**Determination coef. (R^2)**	0.99777					
**Adjusted R^2**	0.99768					
**Standard error**	0.01006					
**n**	27					
**ANOVA TEST**						
	**Degrees of Freedom**	**Sum of Squares**	**Mean of Squares**	**F**	**p-Value**	
Regression	1	1.131	1.131	11184	1.147E-34	
Residual	25	0.003	0.000			
Total	26	1.134				
	**Estimation**	**Std. Error**	**t-Statistic**	**p-Value**	**Lower 99%**	**Upper 99%**
Intercept	-0.00106	0.00357	-0.29820	0.76801	-0.01101	0.00888

Slope	0.00238	0.00002	105.75538	0.00000	0.00232	0.00244

The equation of the calibration line was as follows: y = 0.00238 x – 0.00106 (n= 27), where y represents the absorbance value, and x the concentration of the Polyphenon 60® (μg mL^{-1}). According to statistical analysis by ANOVA, the curves were linear with $p < 0.01$.

Precision. The precision of the method was evaluated by intermediate precision (inter-day), and repeatability (intra-day), and expressed as relative standard deviation (%RSD). Repeatability was determined by analyzing six replicate injections of samples prepared with concentration of 33, 133 and 267 μg mL^{-1} of Polyphenon 60® the same day under the same experimental conditions. The intermediate precision (inter-day precision) of the method was evaluated by analyzing three independent samples (n= 6) on two different days, maintaining all the solutions at 4ºC.

The repeatability (intra-day precision) and the time-different intermediate precision (inter-day precision), expressed as percentage relative standard deviation (% RSD) for Polyphenon 60® at low, medium and high concentration levels are shown in Table S2.

Table 2. Main precision (repeatability and intermediate precision) and robustness results obtained at three different concentration levels: low, medium and high (L/M/H).

Parameter	Obtained value
Response method RSD (M) (%) (*n* = 3)	3.08
Repeatability (intra-day) (*n* = 6)	
RSD (L) (%)	4.25
RSD (M) (%)	1.67
RSD (H) (%)	1.19
Day-to-day repeatability (*n* = 6)	
RSD (L) (%)	0.57
RSD (M) (%)	0.36
RSD (H) (%)	1.21
Robustness (medium)	
RSD (L) (%)	4.87
RSD (M) (%)	1.94
RSD (H) (%)	0.85

For intra-day precision the %RSD values ranged from 1.67 to 4.25. The %RSD of intermediate precision (inter-day) ranged from 0.36 to 1.21. In both cases the %RSDs found are lower than 5% and can therefore be considered acceptable.

The repeatability (intra-day precision) and the time-different intermediate precision (inter-day precision), expressed as percentage relative standard deviation (% RSD) for Polyphenon 60® at low, medium and high concentration levels are shown in Table 3.

Table 3. Main precision (repeatability and intermediate precision) and robustness results obtained at three different concentration levels: low, medium and high (L/M/H).

Parameter	Obtained value
Response method RSD (M) (%) (*n* = 3)	3.08
Repeatability (intra-day) (*n* = 6)	
RSD (L) (%)	4.25
RSD (M) (%)	1.67
RSD (H) (%)	1.19
Day-to-day repeatability (*n* = 6)	
RSD (L) (%)	0.57
RSD (M) (%)	0.36
RSD (H) (%)	1.21

Robustness (medium)	
RSD (L) (%)	4.87
RSD (M) (%)	1.94
RSD (H) (%)	0.85

For intra-day precision the %RSD values ranged from 1.67 to 4.25. The %RSD of intermediate precision (inter-day) ranged from 0.36 to 1.21. In both cases the %RSDs found are lower than 5% and can therefore be considered acceptable.

Accuracy/recovery. Accuracy of the method was determined based on the percentage recovery of known amounts of Polyphenon 60® after analyzing three samples prepared with concentration ratios of 33, 133 and 267 μg mL^{-1}. All samples were prepared in triplicate and the accuracy of the assay was estimated by comparing the found concentration with respect to the concentration added.

Table S3 summarizes the results obtained for accuracy for Polyphenon 60®. Cochran's G tests (p = 0.05) indicate that the variance values obtained at each concentration level are equivalent. Therefore, it can be concluded that the variable concentration does not have a statistically significant influence on the variability of both methods. The statistical Student-t was also found to be below the tabulated values for all the recoveries obtained at different concentrations. These results denote that no statistically significant differences were found between 100% and the recoveries obtained for both methods. Average recoveries are also included.

Table 3. Statistical analysis and accuracy results.

Parameter	Obtained value
Maximum variance	9.2532
$G_{experimental}$	0.5016
$G_{tabulated}$ (p = 0.05, k = 3, n = 3)	0.8709
$t_{experimental}$	0.310
$t_{tabulated}$ (p = 0.05, k = 8, n = 9)	2.306
Average recovery (%)	99.52
RSD (%)	4.620

Selectivity. Selectivity of the method was verified by checking the results with than obtained by a previously validated HPLC method. Briefly, A Waters 2690 Alliance separation module equipped with a Supelcosil LC-18 analytical (5μm, 150 mm x 4,6 mm I.D.; Bellefonte, PA, USA), protected by a C18 (10 μm, 30 mm × 4.6 mm) pre-column guard cartridge, and coupled with a Waters 996 photodiode array detector (Waters, Milford, MA, USA) were used in the analysis. The analysis was carried out at 20 ºC. Compounds were eluted under gradient in water (0.1% formic acid)/methanol (0.1% formic acid) in which the concentration of water varied as follows: 0 min, 80%; from 0 to 15 min, decreased to 50%; from 15 to 25 min, 50%. The flow rate was 1 mL min^{-1}, the detection of compounds was achieved at 280 nm, and injection volume was 20 μL. Quantification was achieved using Polyphenon 60® as standard and confirmed with either caffeine or catechins alone as standard, results not included.

Standard solutions of Polyphenon 60® at low, medium and high concentration levels and green tea extracts solutions were analyzed according to the previously validated HPLC method and checked with the herein presented spectrophotometric method. The obtained chromatograms showed clear, compact and well-separated peaks of catechins and good correlations for both methods (results not included).

Sensitivity. Sensitivity of the method was analyzed by calculating the detection limit (LOD) and the quantification limit (LOQ).

The LOD and LOQ obtained were 0.3 μg mL^{-1} and 1.2 μg mL^{-1} for ECG, the catechin at lowest concentration in Polyphenon 60®. These values are adequate for the detection and quantification of these compounds.

Robustness. Robustness was analysed under different experimental conditions such as changes in the buffer composition at the maximum sensitivity pH. In this way, two different pH 3.6 buffers were tested, NaCl 0.28 M, monosodium phosphate 5 mM and 30 mM sodium acetate buffer. The effects of these experimental conditions on the percentage recoveries and repeatability were studied.

Changes in the buffer composition at the maximum sensitivity pH on the percentage relative standard deviation (% RSD) for Polyphenon 60® at low, medium and high concentration levels are 4.87, 1.94 and 0.85 respectively. In the light of the above results, both buffers can therefore be considered acceptable.

Considering the content of gallic acid in Polyphenon 60®, it has been obtained an experimental value of 27.4% of gallic acid in Polyphenon 60® that closely approximates the theoretical one. It is possible that no gallic phenols present in the catechins also react with Fe(III) and are responsible for this small difference.

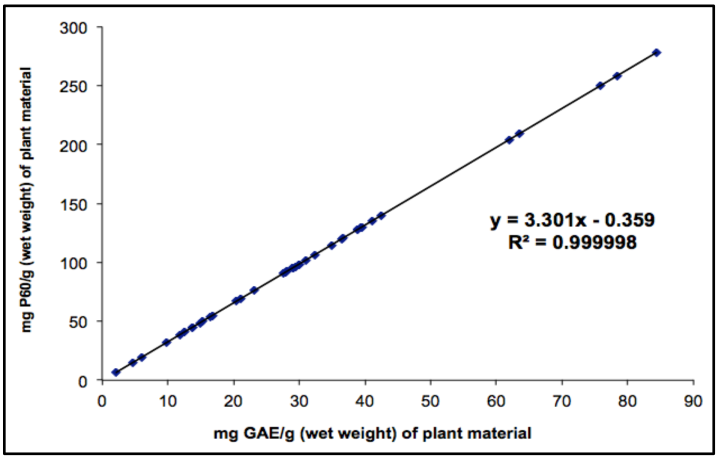

Figure S2. Correlation plot between gallic acid and Polyphenon 60®.

As can be seen in Figure S2 a determination coefficient close to 1 indicates that the observed reactivity is due mostly to the gallic acid in the preparation of Polyphenon 60®.
